# A Prospective Study of Comparing Multi-Gene Biomarker Chip and Serum Carcinoembryonic Antigen in the Postoperative Surveillance for Patients with Stage I-III Colorectal Cancer

**DOI:** 10.1371/journal.pone.0163264

**Published:** 2016-10-04

**Authors:** Yu-Tang Chang, Ming-Yii Huang, Yung-Sung Yeh, Ching-Wen Huang, Hsiang-Lin Tsai, Tian-Lu Cheng, Jaw-Yuan Wang

**Affiliations:** 1 Division of Gastroenterology and General Surgery, Department of Surgery, Kaohsiung Medical University Hospital, Kaohsiung Medical University, Kaohsiung, Taiwan; 2 Graduate Institute of Clinical Medicine, College of Medicine, Kaohsiung Medical University, Kaohsiung, Taiwan; 3 Department of Surgery, Faculty of Medicine, College of Medicine, Kaohsiung Medical University, Kaohsiung, Taiwan; 4 Division of Pediatric Surgery, Department of Surgery, Kaohsiung Medical University Hospital, Kaohsiung Medical University, Kaohsiung, Taiwan; 5 Department of Radiation Oncology, Cancer Center, Kaohsiung Medical University Hospital, Kaohsiung, Taiwan; 6 Department of Radiation Oncology, Faculty of Medicine, College of Medicine, Kaohsiung Medical University, Kaohsiung, Taiwan; 7 Center for Biomarkers and Biotech Drugs, Kaohsiung Medical University, Kaohsiung, Taiwan; 8 Division of Trauma and Critical Care, Department of Surgery, Kaohsiung Medical University Hospital, Kaohsiung Medical University, Kaohsiung, Taiwan; 9 Department of Emergency Medicine, Kaohsiung Medical University Hospital, Kaohsiung Medical University, Kaohsiung, Taiwan; 10 Division of Colorectal Surgery, Department of Surgery, Kaohsiung Medical University Hospital, Kaohsiung Medical University, Kaohsiung, Taiwan; 11 Graduate Institute of Medicine, College of Medicine, Kaohsiung Medical University, Kaohsiung, Taiwan; 12 Division of General Surgery Medicine, Department of Surgery, Kaohsiung Medical University Hospital, Kaohsiung, Taiwan; 13 Department of Biomedical Science and Environmental Biology, Kaohsiung Medical University, Kaohsiung, Taiwan; 14 Research Center for Environment Medicine, Kaohsiung Medical University, Kaohsiung, Taiwan; National Cancer Center, JAPAN

## Abstract

**Background:**

Circulating biomarkers can predict clinical outcomes in colorectal cancer patients. The aim of the study was to evaluate the feasibility of our multigene biomarker chip for detecting circulating tumor cells for postoperative surveillance of stage I–III colorectal cancer patients.

**Materials and Methods:**

In total, 298 stage I–III colorectal cancer patients were analyzed after curative resection between June 2010 and October 2014. During each follow-up, a postoperative surveillance strategy, including ESMO Guidelines Working Group recommendations and the biochip, was used.

**Results:**

After a 28.4-month median follow-up, 48 (16.1%) patients had postoperative relapse. Univariate analysis revealed that the postoperative relapse risk factors were rectal tumor, perineural invasion, elevated preoperative and postoperative serum carcinoembryonic antigen levels, and positive biochip results (all *P* < 0.05). Multivariate analyses revealed that postoperative relapse correlated significantly with elevated postoperative serum carcinoembryonic antigen levels (odds ratio = 4.136, *P* = 0.008) and positive biochip results (odds ratio = 66.878, *P* < 0.001). However, the sensitivity (*P* = 0.003), specificity (*P* = 0.003), positive (*P* = 0.002) and negative (*P* = 0.006) predictive values, and accuracy (*P* < 0.001) of the biochip for predicting postoperative relapse were significantly higher than those of elevated postoperative serum carcinoembryonic antigen levels. Moreover, the median lead time between positive biochip result and postoperative relapse detection was significantly earlier than that between elevated postoperative serum carcinoembryonic antigen level and postoperative relapse detection (10.7 vs. 2.8 months, *P* < 0.001). Furthermore, positive biochip results correlated strongly with lower disease-free survival and overall survival of colorectal cancer patients (both *P* < 0.001).

**Conclusion:**

Compared with conventional serum carcinoembryonic antigen detection, our multigene chip aided more accurate and earlier prediction of postoperative relapse during stage I–III colorectal cancer patient surveillance. In clinical practice, this biochip may facilitate early postoperative relapse diagnosis in colorectal cancer patients.

## Introduction

According to the Ministry of Health and Welfare of Taiwan, since 2006, colorectal cancer (CRC) has become the most common cancer and the third cause of cancer-related death in Taiwan. In 2013, its incidence was 45.1 per 100,000 population, with more than 5,000 deaths per year and an average of 13.1 years of life lost (http://mohw.gov.tw/CHT/DOS/Index.aspx; accessed in March 2016). Although surgical resection is the primary treatment modality for CRC, 33%–50% of CRC patients relapse [1]. More than 90% of relapses occur during the first 5 years following surgery and at a particularly higher rate in the first 2 years. However, CRC-related deaths are majorly attributable to clinical relapse [1]. If the relapse is diagnosed earlier, it may be amenable to resection, leading to a higher rate of resectability and increasing the likelihood of long-term survival [2].

Several surveillance strategies for patients undergoing curative primary CRC resection have been reported. Carcinoembryonic antigen (CEA), an oncofetal antigen, is an extensively used disease relapse marker [3]; however, the utility of serial CEA testing remains uncertain: in 30%–40% of all CRC recurrences, the serum CEA shows unmeasurable elevations [4]. By contrast, transient elevations in CEA levels are observed in patients with resected CRC during adjuvant chemotherapy or immunotherapy. The false-positive rate for elevated serum CEA level detection during follow-up can be as high as 16% [5], unnecessarily increasing the difficulty in diagnosing recurrence and increasing patient anxiety [2]. Therefore, a more powerful tool for early detection of CRC relapse is required.

Studies for identifying novel panels of multiple molecular and biochemical markers usable for more precisely defining prognosis and predicting of adjuvant treatment benefits in CRC have been reported [6]. Reports have described the detection of circulating tumor cells (CTCs) in the peripheral blood of CRC patients; this method has major prognostic and therapeutic implications [7–10]. Our recently developed membrane array-based multigene biomarker assay can detect CTCs in the peripheral blood of CRC patients; this is a rational approach for the surveillance of postoperative CRC patients [6,11–15]. However, a detailed prospective comparative study regarding the diagnostic accuracy of the biomarker chip and serum CEA level detection is required. In the present study, we prospectively analyzed both the biomarker chip and serum CEA level detection periodically after curative resection in Union for International Cancer Control (UICC)/American Joint Committee on Cancer stage I–III CRC patients and identified whether the biochip was more efficient for their postoperative surveillance.

## Materials and Methods

### Patients

This prospective study was conducted by a surgical team in a single institution between June 2010 and February 2016. During June 2010 to October 2014, 331 patients were diagnosed with stage I–III CRC. Of these, 33 were excluded: 16 with a <1-year follow-up before death and 17 with other malignancies. Finally, 298 patients were enrolled after radical curative resection for primary CRC tumor: 82, 102, and 114 stage I, II, and III patients, respectively. The clinicopathological characteristics of these patients are listed in Table 1. The clinical stages and pathological features of the primary tumors were defined according to the seventh edition of the UICC tumor—node—metastasis (TNM) staging system [16].

**Table 1:** Clinicopathological features of 298 colorectal cancer patients.

Gender (male/female)	168/130
Age (year)	
Median	64.21
Mean ± SD	64.4±11.3
Maximum tumor size≧5cm	78 (26.2%)
Location (rectum/colon)	77/221
Depth of tumor invasion T (1/2/3/4)	44/67/167/20
Lymph node metastasis N (0/1/2)	184/74/40
Histology (WD/MD/PD)	42/244/12
TMN stage (I/II/III)	82/102/114
Vascular invasion	86 (28.9%)
Perineural invasion	61 (20.5%)
Abnormal preoperative CEA level	98 (32.9%)
Abnormal postoperative CEA level	71 (23.8%)
Postoperative relapse	48 (16.1%)
Positive biomarker chip	62 (20.8%)
Mortality	26 (8.7%)
Follow up (month)	
Median, range	28.4, 3.0–61.3
Mean ± SD	29.0±9.7

### Ethics Statement

The study protocol was approved by the Institutional Review Board of Kaohsiung Medical University Hospital (KMUHIRB-950326 & KMUHIRB-2012-03-02(II)). Written informed consent was obtained from the patients, and all clinical investigations were conducted according to the principles expressed in the Declaration of Helsinki. The patients also consented to the publication of the clinical details.

### Follow-up

According to the clinical practice guidelines recommended by European Society for Medical Oncology Guidelines Working Group [1], postoperative surveillance during each follow-up comprised medical history-taking, physical examination, and laboratory studies including serum CEA levels. Abdominal ultrasonography or computed tomography (CT) was performed every 6 months, and chest plain radiography examinations and total colonofiberscopy were performed once a year. Furthermore, elevated CEA levels were defined when two consecutive CEA levels at a 3-month interval of regular follow-up were >5 ng/mL. In the case of elevated CEA or a positive multigene biomarker chip, high resolution MRI or contrast enhanced CT of the liver was performed before the annual follow-up. Patients were followed every 3 months in the first 3 years and at 6-month intervals thereafter. All patients were followed until death or February 2016. The development of recurrent or metastatic lesions was defined as postoperative relapse.

CTCs in the peripheral blood were detected using our previously constructed multigene biomarker chip with serial CEA assays at each follow-up [7, 12–15]. Additional 4 mL samples of peripheral blood were obtained for total RNA isolation. To prevent contamination by epithelial cells, peripheral blood samples were obtained through a catheter inserted into a peripheral vessel, and the first 5 mL of blood was discarded. Sample acquisition and subsequent use were approved by the institutional review board of the hospital.

### Serum CEA Level Detection

Serum CEA levels were determined from additional 3 mL peripheral blood samples by using an enzyme immunoassay test kit (DPC Diagnostic Product Co., Los Angeles, CA, USA), with the upper limit of 5 ng/mL defined as normal, in accordance with the manufacturer instructions.

### Gene Selection and Oligonucleotide Design

The authors used a method combining suppression subtractive hybridization and cDNA microarray chips to investigate changes in all genes involved in the carcinogenic pathway from colorectal adenomatous polyps to colorectal cancer [17]. 71 genes specific for colorectal cancer as diagnostic markers were successfully identified and the patents were obtained in Taiwan (No. I278519), the USA (No. US 7575928), and the European Union (No. 04 003 301.1). In order to implement the clinical application of specific gene groups, the oligonucleotide sequences of top 19 highly overexpressed target genes of the 71 genes were selected (Table 2), as described previously [18–20]; they were designed using the Oligo Explorer software program (Gene Link, Inc. New York, USA).

**Table 2:** Oligonucleotide sequences of 19 target genes.

Gene	oligonucleotide sequences (5'→ 3')
PSG2	CTCGGAAACTTTTGGTGGCTGGGCTTCAATCGTGACTTGGGCAGT
ELAVL4	TTGCCCCTGTTTGCATGGGAGAAGGACAGTTTCTGTTGTTGCTGG
TK1	CAGGGAGAACAGAAACTCAGCAGTGAAAGCCGCAGAGGGGAAGAA
UBE2C	AACTTCACTGTGGGCGCATTGTAAGGGTAGCCACTGGGGAACTCT
PDE6D	TGCCAGAGTATCTTCCCTGTCTCAGCATCCCGAAGGTTCATCCAA
PSAT1	TTGACCTTGAATCAACAGCCGCTGAACCCAGGAGACCCCACAGAT
CHRNB1	TAGGGTCCCAACGCTGGTGAAGATGATGAAAGTCCACAGGAAGAG
CEA	ATCCTGCATCGTTCCTTTTGACGCTGAGTAGAGTGAGGGTCATGT
BMI1	CGAGGTCTATTGGCAAAAGAAGATTGGTGGTTACCGCTGGGGCTG
CAP2	ACATGGCGGAGCCCTTTTGTAATTGCTTCTCCCTGGTTAAGTTGG
MMP13	AAAGTGGCTTTTGCCGGTGTAGGTGTAGATAGGAAACATGAGTGC
OLFM4	AGCAGGTGCCTCATCTACAGATCCTTCTGGGATTTATTTGCCATG
PTTG1	TATCTATGTCACAGCAAACAGGTGGCAATTCAACATCCAGGGTCG
MYC	AGTGACTGTCCAGTTTTGAGAAGCGTCTAGCAAGTCCGAGCGTGTTCAAT
MET	CCCGAGTTCTTTCTATTGATGCGTTCATGCTCTTGACCCTGGTAG
MUC1	CCTGGGGTAGAGCTTGCATGACCAGAACCTGTAACAACTGTAAGCACTGT
HMGB1	ATTGCAGCCTATCACTAACCCTGCTGTTCGCTTGCATGTATCTTG
hTERT	AGGGGTGAACAATGGCGAATCTGGGGATGGACTATTCCTATGTGG
BIRC5	CTCTAACCTGCCATTGGAACCTCACCCATAGCCCAGAAGCCTCAT
*Oryza sativa*	CTCGGTAACCTCTATTCCTCTACACCCTCGACCTCACCAACACCAGCCT
*β-actin*	ATGCTCGCTCCAACCGACTGCTGTCACCTTCACCGTTCCAGTTTT

### Multigene Biomarker Chip Preparation

The 19 synthesised oligonucleotides were dissolved in distilled water to a concentration of 100 mM and then applied to a BioDOT AD1500 nanoliter dispense system (BioDot, Inc., Irvine, CA, USA), which blotted each oligonucleotide solution sequentially on a Nytran SuperCharge nylon membrane (Schleicher and Schuell, Dassel, Germany) in triplicate. The oligonucleotides were then crosslinked to the membrane by using a UV Stratalinker 1800 (Stratagene, La Jolla, CA, USA). Each spot contained 20 ng of PCR-amplified DNA derived from sequence-verified cDNA clones. DMSO was also dispensed onto the membrane as a blank control.

### Detection of Multigene Expression on the Biomarker Chip

A GeneCling Enzymatic Gene Chip Detection Kit (CaryGene Biotechnology Co., Ltd., Kaohsiung, Taiwan) was used as follows: Beads first were added for RNA extraction; RT buffer and RT enzyme were then added to synthesize cDNA. Next, Biotin Mix Label was added to the cDNA for biotin-labelling probe synthesis (probe synthesis). The labelled cDNA was then hybridised with the prepared biomarker chip at 42°C for 6–17 h. Wash buffer was then used to wash off the nonhybridized probes, followed by the addition of Streptavidin-AP—NBT/BCIP mixture and incubation at 37°C for a color reaction. Finally, after coloration, the biochips were completely dried, and the software was used to analyze the colorimetric values of the individual genes; these values were translated to indicate the relative intensities of the various gene expressions.

### Biomarker Chip Intervention

**Fig 1:**
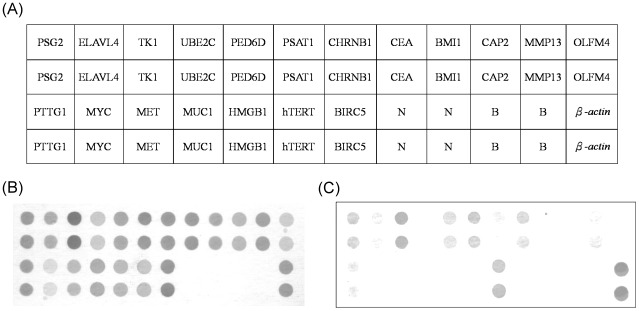
(A) Schematic representation of the colorectal cancer biomarker chip evacuated using the weighted enzymatic chip array method with the 19 candidate genes, a positive control (β-actin), a negative control (*Oryza sativa*), and a blank control (double distilled water). Oligonucleotide fragments were blotted on the membranes in triplicate, and the expression levels of each gene spot were quantified and then normalized on the basis of the color density of a reference gene (β-actin). (B) Positive biochip result. (C) Negative biochip result.

Subsequent quantification analysis of the intensity of each spot was carried out using AlphaEase^®^ FC software (Alpha Innotech Corp., San Leandro, CA, USA). Spots consistently carrying a factor of two or more were considered to be differentially expressed. A deformable template extracted the gene spots and quantified their expression levels by determining the integrated intensity of each spot after background subtraction (Fig 1). The fold ratio of each gene was normalized on the basis of the reference gene (β-actin) density, as follows: spot intensity ratio = mean intensity of the target gene/mean intensity of β-actin. Fig 1 provides the schematic representation of the membrane array with 19 candidate genes, one positive control (β-actin), one negative control (Oryza sativa sequence), and the blank control (dd water). If the gene presented a color density >2-fold higher than the positive control (β-actin) did, the result was defined as positive, whereas if the density was <2-fold higher than that of the positive control, the result was defined as negative. Each overexpressed spot was then multiplied by the respective weighted values ranging from 1 to 4, according to the principle described previously [18–22].

### ROC Curve and Determination of Cutoff Levels of the Multigene Biomarker Chip

The optimal cutoff value of the multigene biomarker chip was determined according to a prior study [18]. Receiver operating characteristic (ROC) curve analysis was performed on 557 participants, including 298 CRC patients (not the enrolled patients in the present study) and 259 normal individuals. Based on the calculated cutoff value, the expression of the biomarker chip was defined as either positive or negative. The optimal cutoff value and AUC were 23.5 and 0.980 (95% CI, 0.971–0.989), respectively. When the total score was ≥ 24 by two consecutive analysis, the biomarker chip results were defined as positive.

### Data Collection and Statistical Analysis

Student *t* and chi-squared tests were used to compare continuous and categorical descriptive variables, respectively, between relapsed and non-relapsed patients. Univariate and multivariate logistic regression analyses were also used to examine the factors influencing the postoperative relapse. The cumulative disease-free survival (DFS) and overall survival (OS) rates were calculated using the Kaplan—Meier method, and the differences in the rates were analyzed using the log-rank test. Results are expressed as the mean with standard deviation or effect and 95% CI where appropriate. A P value of <0.05 denoted statistical significance. All data were analyzed using SPSS (version 19.0; SPSS, Inc., Chicago, IL, USA).

## Results

### Descriptive Data

The median follow-up time was 28.4 months (range, 3.0–61.3 months). Of the 298 patients, 168 were men (56.4%). The average age was 64.4 ± 11.3 years (range, 20–91 years). Regarding tumor histology, 42 (14.1%) were well-differentiated carcinomas, 244 (81.9%) were moderately differentiated, and 12 (4.0%) were poorly differentiated. Forty-eight (16.1%) patients had postoperative relapse and 26 (8.7%) died. Of the 298 CRC patients, 62 (20.8%) had a total biomarker chip score higher than the cutoff value. Of 48 relapsed patients, 42 (87.5%) showed positive biochip results prior to relapse. The positive biochip results were significantly associated with postoperative relapse (P < 0.001; Table 3). The clinicopathological characteristics of all 298 CRC patients are listed in Table 1. The raw data from the multigene biomarker chip may compromise the privacy of study participants and may not be shared publicly. Data are available upon request to the authors.

**Table 3:** Comparison between non-relapsed and relapsed colorectal cancer patients.

	Non-relapse N = 250	Relapse N = 48	*P*
Gender (Male/Female)	144/106	24/24	0.331
Age (year)	64.2±11.0	65.3±12.8	0.546
Maximum tumor size≧5cm	66 (26.4%)	12 (25%)	0.840
Location (rectum/colon)	58/192	19/29	0.018
Depth of tumor invasion	96/154	15/33	0.348
T (1+2/3+4)			
Lymph node metastasis	157/93	27/21	0.392
N (0/1+2)			
Histology (WD+MD/PD)	214/9	45/3	0.392
TNM stage (I-II/III)	157/93	27/21	0.392
Vascular invasion	68 (27.2%)	18 (37.5%)	0.149
Perineural invasion	45 (18.0%)	16 (33.3%)	0.016
Abnormal preoperative CEA level	72 (28.8%)	26 (54.2%)	0.001
Abnormal postoperative CEA level	42 (16.8%)	29 (60.4%)	<0.001
Positive biomarker chip	20 (8.0%)	42 (87.5%)	<0.001
Mortality	6 (1.6%)	20 (29.5%)	<0.001
Follow up (month)			
Median, range	29.6, 7.3–59.8	24.1, 3.0–61.3	0.058
Mean ± SD	29.5±9.5	26.6±10.6	

### Univariate and Multivariate Analyses

During the follow-up period, 29 of 221 (13.1%) colon cancer patients and 19 of 77 (24.7%) rectal cancer patients showed postoperative relapse. In comparison with the patients without postoperative relapse, rectal neoplasms (*P* = 0.018), perineural invasion (*P* = 0.016), elevated preoperative serum CEA levels (*P* = 0.001), elevated postoperative serum CEA levels (*P* < 0.001), and positive biochip results (*P* < 0.001) were more frequently noted in the patients with relapse (Table 4). However, sex, age, tumor size, tumor invasion depth, lymph node metastasis, histology, TNM stage, vascular invasion, and follow-up duration did not differ significantly between the studied groups (all *P* > 0.05).

**Table 4:** Factors influencing the relapse estimated by univariate and multivariate logistic regression analyses.

	Univariate regression		Multivariate regression	
	Odds ratio (95% CI)	*P*	Odds ratio (95% CI)	*P*
Maximum tumor size≧5cm	0.929 (0.456, 1.893)	0.840	-	-
Location (rectum/colon)	2.169 (1.134, 4.149)	0.019	1.566 (0.564, 4.348)	0.389
Depth of tumor invasion	1.371 (0.708, 2.657)	0.349	-	-
T (1+2/3+4)				
Lymph node metastasis	1.313 (0.703, 2.454)	0.393	-	-
N (0/1+2)				
Histology (WD+MD/PD)	1.785 (0.465, 6.851)	0.398	-	-
TNM stage (I+II/III)	1.313 (0.703, 2.454)	0.393	-	-
Vascular invasion	1.606 (0.841, 3.068)	0.152	-	-
Perineural invasion	2.278 (1.152, 4.502)	0.018	2.181 (0.716, 6.644)	0.170
Abnormal preoperative CEA level	2.922 (1.556, 5.488)	0.001	2.538 (0.885, 7.277)	0.083
Abnormal postoperative CEA level	7.559 (3.880, 14.724)	<0.001	4.136 (1.455, 11.755)	0.008
Positive biomarker chip	80.500 (30.523, 212.309)	<0.001	66.878 (23.229, 192.548)	<0.001

95% CI: 95% confidence interval

In the multivariate logistic regression analysis, elevated postoperative serum CEA levels (OR = 4.136, 95% CI: 1.455–11.755; *P* = 0.008) and positive biochip results (OR = 66.878, 95% CI: 23.229–192.548; *P* < 0.001) were revealed to be independent predictors for postoperative relapse (Table 5). However, tumor location (rectum or colon), perineural invasion, and preoperative CEA elevation did not differ significantly between the studied groups (all *P* > 0.05).

**Table 5:** Sensitivity, specificity, postitive predictive value, negative predictive value, and accuracy of postoperative serum CEA level and biomarker chip.

	Abnormal postoperative CEA level (95% CI)	Positive biomarker chip (95% CI)	*P*
Sensitivity	60.4%	87.5%	0.003
	(45.3%-74.2%)	(74.8%-95.3%)	
Specificity	83.2%	92.0%	0.003
	(78.0%-87.6%)	(87.9%-95.1%)	
Positive predictive value	40.8%	67.7%	0.002
	(29.3%-53.2%)	(54.7%-79.1%)	
Negative predictive value	91.6%	97.5%	0.006
	(87.2%-94.9%)	(94.6%-99.1%)	
Accuracy	79.5%	91.3%	<0.001
	(74.9%-84.1%)	(88.1%-94.5%)	

95% CI: 95% confidence interval

### Sensitivities, Specificities, and Accuracies of Postoperative Serum CEA levels and Biomarker Chip for Predicting Postoperative Relapse

Positive biochip results with elevated postoperative serum CEA levels for predicting postoperative relapse were thoroughly compared (Table 5). The biomarker chip demonstrated higher sensitivity (biochip: 87.5%, CEA: 60.4%; *P* = 0.003), specificity (biochip: 92.0%, CEA, 83.2%; *P* = 0.003), positive predictive value (biochip: 67.7%, CEA: 40.8%; *P* = 0.002), negative predictive value (biochip: 97.5%, CEA: 91.6%; *P* = 0.006), and accuracy (biochip: 91.3%, CEA: 79.5%; *P* < 0.001) than postoperative serum CEA levels did. Therefore, our multigene biomarker chip would be a more accurate tool for predicting postoperative relapse than postoperative serum CEA is.

In clinical practice, the two independent tests can be combined to be more confident of the diagnosis. The combined specificity becomes 1-(1–0.832)×(1–0.92) = 1–0.001344 = 0.998656 = 99.8656%. The combined sensitivity becomes = 0.604×0.875 = 0.5285 = 52.85%. The combined specificity of 99.87% allowed us to rule in the diagnosis: until proved otherwise, this patient had postoperative relapse.

The diagnostic/prognostic values of the biochip and postoperative CEA were evaluated according to different clinical features and shown in Table 6. The positive biochip results showed prominent association with rectal tumor (*P* = 0.009), perineural invasion (*P* = 0.010), postoperative relapse (*P* < 0.001) and mortality (*P* < 0.001).

**Table 6:** Clinical features associated with diagnostic/prognostic values of postoperative CEA and the biochip.

	Postoperative CEA (+/-)	Biochip (+/-)	*P*
Maximum tumor size			0.690
≧5cm	20/58	15/63	
<5cm	51/169	47/173	
Location			0.009
Rectum	22/55	24/53	
Colon	49/172	38/183	
Depth of tumor invasion			0.361
T (1+2)	23/88	20/91	
T (3+4)	48/139	42/145	
Lymph node metastasis			0.934
N (0)	42/142	38/146	
N (1+2)	29/85	24/90	
Histology			0.069
WD+MD	68/218	57/229	
PD	3/9	5/7	
TNM stage			0.934
I+II	42/142	38/146	
III	29/85	24/90	
Vascular invasion			0.328
Positive	24/62	21/65	
Negative	47/165	41/171	
Perineural invasion			0.010
Positive	12/49	20/41	
Negative	59/178	42/195	
Postoperative relapse			<0.001
Yes	29/19	42/6	
No	42/208	20/230	
Mortality			<0.001
Yes	15/11	19/7	
No	56/216	43/229	

### Multigene Biomarker Chip versus Postoperative Serum CEA Levels for Predicting Postoperative Relapse and Clinical Outcomes

**Fig 2:**
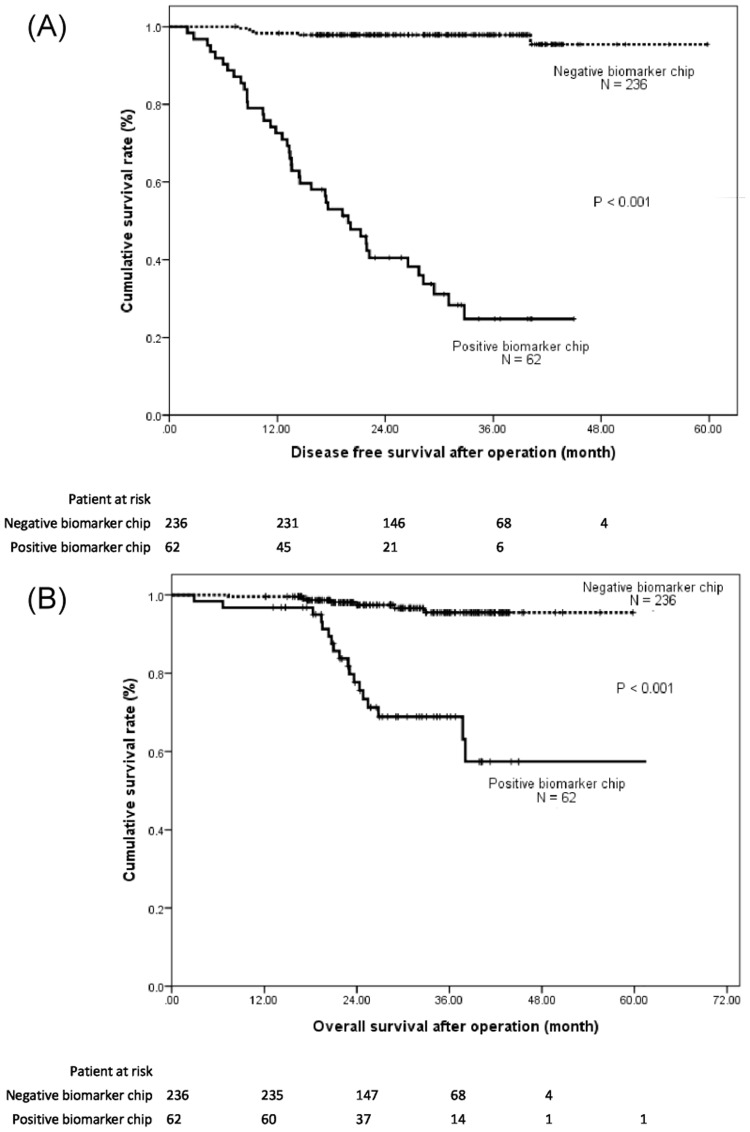
(A) Cumulative disease-free survival (DFS) and (B) overall survival (OS) rates of the 298 colorectal cancer patients calculated using the Kaplan—Meier method. Positive biochip results correlated strongly with lower DFS and OS rates of colorectal cancer patients (both *P* < 0.001).

In postoperative surveillance, both multigene biomarker chip analysis and CEA assays were performed at each follow-up. Of the 48 relapsed patients, 42 (87.5%) showed positive biochip results and a median lead time from detection of 10.7 months (11.0 ± 7.3 months; Table 7). However, only 29 (60.4%) relapsed patients had elevated postoperative serum CEA levels, and the median lead time was 2.8 months (3.4 ± 2.8 months). The median lead time between the positive biochip results and subsequent postoperative relapse detection was considerably earlier than that between the elevated postoperative serum CEA levels and postoperative relapse detection (*P* < 0.001). The median DFS rate was significantly lower among patients with positive biochip results than among patients with negative biochip results (20.4 vs. 48.0 months, *P* < 0.001; Fig 2A). The cumulative DFS rate at the end of the study was 95% and 26% for the patients with negative and positive biochip results, respectively. Similarly, the median OS rate was significantly lower among the patients with positive biochip results than among those with negative biochip results (*P* < 0.001; Fig 2B). The cumulative proportion OS rate at 48 months was 96% and 51% for those with negative and positive biochip results, respectively.

**Table 7:** Comparison of the expression prior to the diagnosis in 48 relapsed patients.

	Postoperative CEA level	Biomarker chip	*P*
Positive result N (%)	29 (60.4)	42 (87.5)	0.003
Lead-time (month)			<0.001
Median (Range)	2.8 (0.5–11.0)	10.7 (0.5–30.7)	
Mean ± SD	3.4 ± 2.8	11.0 ± 7.3	

## Discussion

Postoperative surveillance of CRC patients facilitates early diagnosis of disease relapse, which may then be surgically or medically treated. Of the numerous reported follow-up strategies, serial CEA monitoring is the most sensitive for detecting recurrence compared with history-taking, physical examination, liver function tests, abdominal sonography, chest radiography, and colonofiberoscopy; it remains one of the most crucial postoperative work-ups [23–25]. In the literature, the estimated sensitivity of serum CEA for detecting relapsed disease in patients with completely resected CRC is 58%–89%, with a 1.5–6.0-month lead time between serum CEA level elevation and recurrence detection [26–29]. In the present study, the sensitivity and specificity of elevated serum CEA level detection were 60.4% and 83.2%, with a median lead time of 2.8 months between serum CEA level and relapse detection, consistent with the previous reports. However, the sensitivity and specificity of relapse detection depends largely on the definition of elevated serum CEA levels (cutoff value). The higher the cutoff value for the elevated serum CEA levels is, the higher (lower) the specificity (sensitivity) would be [27, 30].

Elevated preoperative serum CEA levels indicate a poor prognosis and are correlated with a reduced OS after surgical resection [27, 31–32]. In the present study, the sensitivity, specificity, positive predictive value, negative predictive value, and accuracy of elevated preoperative serum CEA levels for predicting postoperative relapse were 54.1%, 71.2%, 26.5%, 89.0%, and 68.5%, respectively. In addition, the elevated preoperative serum CEA levels were more frequent in patients with relapse than in those without relapse (*P* = 0.001). However, in the multivariate analysis, the postoperative relapse was not associated with elevated preoperative serum CEA levels (*P* = 0.083). Of all clinicopathological features, well known factors, such as depth of invasion and lymph node metastasis, did not affect the recurrence rate in univariate analysis, and the relatively short follow-up period may in part be responsible for it. The elevated postoperative serum CEA levels and positive biochip results were the only two independent predictors of postoperative relapse (P = 0.008 and P < 0.001, respectively). Of the two independent predictors of relapse, our multigene biomarker biochip was more accurate than postoperative serum CEA levels (91.3% vs. 79.5%, *P* < 0.001). Moreover, the median lead time between positive biochip results and relapse detection was 10.7 months, considerably earlier than that between elevated postoperative serum CEA levels and relapse detection (2.8 months, *P* < 0.001). The detection of overexpressed molecular biomarkers on the biochip may facilitate earlier detection of relapsed disease and enable physicians to select early therapeutic strategies.

A weighted enzymatic chip array (WEnCA) platform is a sensitive technique for detecting activated *KRAS* from the peripheral blood in patients with various malignancies [18,21,22]. The selection of the target gene and modification of the weighted values for the corresponding genes contribute the most to the accuracy in clinical applications. To reduce the false-negative detection CTCs when predicting postoperative relapse, cDNA of multiple biomarkers from the peripheral blood were analyzed at each follow-up; the biomarker chips of the CRC patients with subsequent relapse showed that most of the gene spots expressed more prominently with time.

In the present study, the specificity (92.0%) and accuracy (91.3%), but not the sensitivity (87.5%), of the biomarker chip were similar to those reported in previous studies that used the WEnCA platform [18–22]. The false-positive rate of the biomarker chip in early prediction of postoperative relapse was 32.3%; nevertheless, postoperative serum CEA levels showed a higher false-positive rate (59.2%). These rates may have been low because of using unadjusted weighted value and limited follow-up period. Therefore, to obtain an acceptable low false-positive rate, individual weighted values of each gene should be further investigated on the WEnCA platform.

Of the 242 patients with negative biochip results, 1 and 4 stage II and III patients relapsed during the follow-up period, respectively. In the biomarker chip of all 5 patients, some gene spots indicating metastatic potential lacked overexpression. Such negative biochip results may be attributable to the histological dedifferentiation or heterogeneity of relapsed tumor or the relapse caused by provocative agents in the environment [33].

In the present study, 9, 18, and 21 stage I, II, and III patients demonstrated postoperative relapse (*P* = 0.328) and a median DFS of 21.3, 12.8, and 9.3 months (*P* = 0.081), respectively. The positive predictive value of the biomarker biochip was more favourable than that of postoperative serum CEA levels (67.7% vs. 40.8%, *P* = 0.002). However, of the 62 patients with a positive biomarker biochip result, 5, 7, and 8 stage I, II, and III patients remained relapse-free until the end of the follow-up period, respectively.

In the present study, we confirmed that our constructed multigene biomarker chip is feasible for the accurate early prediction of postoperative relapse in stage I–III CRC patients. The biomarker chip may be used periodically in clinical practice for postoperative surveillance to improve early detection. However, a multicenter trial for CRC patients with a longer follow-up duration is required in order to confirm the long-term effectiveness.
